# Adverse childhood experience and non-suicidal self-injury in male prisoners: a chain mediating effect through sense of security and emotional problems

**DOI:** 10.3389/fpsyg.2026.1729297

**Published:** 2026-01-30

**Authors:** Xianghui Lai, Jue Deng, Yuanhua Ou, Zezheng Huang, Mingquan Zhang

**Affiliations:** 1 Department of Basic Courses, Fujian Police College, Fuzhou, China; 2 Cognitive Neuroscience and Abnormal Psychology Laboratory, Department of Penalty Execution, Fujian Police College, Fuzhou, China; 3 Department of Investigation, Fujian Police College, Fuzhou, China

**Keywords:** adverse childhood experiences, emotional problems, male prisoners, non-suicidal self-injury, sense of security

## Abstract

**Introduction:**

Non-suicidal self-injury (NSSI) is highly prevalent among prisoners and poses substantial challenges to prison management. This study examined the mechanisms linking adverse childhood experiences (ACEs) to NSSI among male prisoners, with a focus on the serial mediating roles of sense of security and emotional problems.

**Methods:**

A sample of 1,205 male inmates from two Chinese prisons completed self-report measures assessing ACEs, sense of security, emotional problems, and NSSI. Responses from 398 incarcerated individuals were included in the subsequent analysis due to their engagement in NSSI behaviors.

**Results:**

The use of structural equation modeling revealed a significant association between ACEs and NSSI among prisoners, with the model providing robust support for the hypothesized predictive pathway. Sense of security and emotional problems sequentially mediated the relationship between ACEs and NSSI. Specifically, ACEs were associated with a diminished sense of security, which was in turn linked to more severe emotional problems; collectively, these factors predicted an increased risk of NSSI. While ACEs exhibited both direct and indirect associations with NSSI behaviors, its relationship with NSSI functions was fully mediated by the pathway involving sense of security and emotional problems.

**Discussion:**

These findings highlight the importance of addressing early trauma, enhancing perceived security, and improving emotional regulation via correctional mental health interventions to reduce NSSI among incarcerated individuals.

## 1 Introduction

Non-suicidal self-injury (NSSI) refers to self-inflicted damage to body tissues without suicidal intent and for purposes not recognized as socially or culturally acceptable (Klonsky, 2011). Common forms of NSSI include cutting the skin, burning, pulling hair, and other forms of self-inflicted harm (Klonsky, 2007). The prevalence of NSSI among incarcerated individuals is significantly higher than in the general population (Favril et al., 2020; Gu et al., 2023; Morales and Guarnero, 2014; Stijelja and Mishara, 2022). NSSI is significantly linked to suicide risk, with half of the prisoners who die by suicide having a history of NSSI (Favril et al., 2020; Humber et al., 2013). Among prisoners, 53% of suicides occur within 1 month of engaging in NSSI (Hawton et al., 2014). NSSI impacts the correctional processes of prisoners and imposes negative consequences for fellow inmates and correctional personnel, including prison guards, posing huge challenges to prison management (Smith and Power, 2014; Smith et al., 2019). However, it is difficult to predict which prisoners might engage in NSSI due to its inherent concealment and abrupt onset (Dixon-Gordon et al., 2012). To facilitate early intervention, it is essential to examine patterns of NSSI among prisoners and potential predictive pathways (Favril et al., 2020; Martin et al., 2014). Despite this need, research into the mechanisms and potential predictors of NSSI in prisoners remains insufficient due to the difficulty of accessing incarcerated individuals and the complexity of factors underlying NSSI.

Currently, adverse childhood experiences are widely recognized as a variable associated with NSSI and may serve as a critical distal risk factor linked to its development (Laporte et al., 2023; Seibert, 2012; Van Heeringen, 2018). Adverse childhood experiences (ACEs), also known as childhood adversities, is used to describe a range of stressful and potentially traumatic events that children can be exposed to while growing up (Berens et al., 2017; Ford et al., 2020). These experiences include, but are not limited to, exposure to domestic violence, physical or emotional abuse, neglect, parental mental illness, and household substance or alcohol misuse, as well as violence from peers, communities, or larger societal groups (World Health Organization, 2018). Such events place children in a state of helplessness, disrupting typical coping mechanisms (Browne and Winkelman, 2007), and are associated with enduring and profound adverse effects on individuals’ physical and mental health (Wren et al., 2025). ACEs are considered a key factor contributing to NSSI among prisoners (Gu et al., 2021; Hariz et al., 2025; Li et al., 2024; Takahashi et al., 2022). Hariz et al. (2025) studied male prisoners in Tunisia and found that, as the frequency of ACEs increased, the risk of self-directed violence among inmates was significantly higher. Takahashi et al. (2022) demonstrated that, among a group of methamphetamine-abusing incarcerated individuals in Japan, ACEs were associated with suicidal ideation and NSSI. Although these authors have highlighted the association between ACEs and NSSI among incarcerated individuals, the underlying mechanisms and effect pathways through which ACEs influence NSSI in this population remain unclear. Therefore, further investigation into the intermediate variables linking ACE and NSSI among prisoners is warranted.

One potentially indirect influencing factor is a sense of security, a trait-like variable associated with ACEs (Deng et al., 2024; Liao et al., 2014; Noonan and Pilkington, 2020; Sokar et al., 2023). The sense of security is considered as the individual’s basic perception of the possibility of threat and menace in real life (Bar-Tal and Jacobson, 1998). As Maslow et al. (1945) pointed out, the sense of security is a fundamental human need and a prerequisite for mental health. It reflects a relatively stable tendency to process information about control in circumstances and interpersonal contexts, encompassing two core dimensions: perceived certainty in control and interpersonal security (Cong and An, 2004). Authors of previous studies have found a correlation between insecurity and mental disorders, including depression and suicide (Blomqvist et al., 2022; De Witte et al., 2016). A sense of security in adulthood gradually develops from early childhood attachment (Liao et al., 2014) and is negatively associated with ACEs, particularly experiences of abuse or neglect (Noonan and Pilkington, 2020; Sokar et al., 2023). Previous research has found that individuals who experienced abuse or neglect during childhood tend to exhibit altered social information processing, which is characterized by interpreting external stimuli as threatening and is reflected in a diminished sense of security (Dodge et al., 1995). Wang et al. (2025) demonstrated the predictive effect of a sense of security on suicide risk for male prisoners. Therefore, we hypothesize that a sense of security is associated with both ACEs and NSSI among male prisoners and further suggest that a sense of security may serve as a mediator between ACEs and NSSI.

Moreover, since imprisonment itself is a significant stressor, we suggest that, in accordance with the stress-diathesis model (Hankin et al., 2005; Rubinstein, 1986; Van Heeringen, 2018), it is necessary to examine proximal risk factors for NSSI during incarceration. Therefore, negative emotions can be considered another mediating variable associated with NSSI. Lundh et al. (2025) found a significant correlation between anxiety symptoms and NSSI, suggesting that clinical screenings should be used to address the intersection of bodily experiences and anxiety. Furthermore, ACEs and emotional problems in adulthood are closely related (Guo et al., 2025; Pettersson et al., 2025). Jones et al. (2025) found that individuals with higher ACE scores exhibited a significantly higher incidence of negative emotional problems. Dendrinos et al. (2025) found that individuals with a history of ACEs were more likely to exhibit anxiety, depression, and gut-brain axis-related symptoms in adulthood. Incarcerated prisoners experience various emotional problems, including anxiety, depression, and irritability, with anxiety being one of the most prevalent negative emotions within this population (Lv et al., 2020). Existing researchers have indicated that emotional problems, including difficulties in emotional regulation, are correlated with NSSI in male prisoners (Taja et al., 2025). Gu et al. (2021) and Gu et al. (2023) proposed that NSSI among male prisoners can be predicted by difficulties in regulating emotions, particularly the cognitive reappraisal of emotion. Therefore, emotional problems appear to be another indirect variable linking ACEs with NSSI in prison inmates. Since emotional problems are complex and encompass multiple dimensions—such as depression, anxiety, and irritability—a composite scale was employed rather than a unidimensional one for assessment.

In summary, the purpose of this study is to examine the mediating role of male prisoners’ sense of security and emotional problems in the association between ACE and NSSI. We hypothesize that a sense of security and emotional problems will serve as sequential mediators in this relationship, primarily based on the following three theoretical grounds.

First, ACEs are associated with a diminished sense of security in individuals (Liao et al., 2014; Noonan and Pilkington, 2020; Sokar et al., 2023). Authors of longitudinal studies have found that secure attachment during childhood serves as the foundation for developing psychological security, while early abuse, neglect, or disorganized attachment are significantly associated with insecurity in adulthood and an increased risk of mental illness (Sroufe, 2005). Authors of brain imaging studies have demonstrated that ACEs are associated with lasting structural alterations in brain regions such as the amygdala, anterior cingulate cortex, and hippocampus; all areas that are critically involved in safety perception, threat appraisal, and emotional regulation (Teicher and Samson, 2016). These neurophysiological mechanisms provide further insights into how ACEs can disrupt an individual’s perception and interpretation of safety at a physiological level.

Second, the absence of a sense of security is closely associated with emotional problems (Blomqvist et al., 2022; De Witte et al., 2016). According to proponents of emotion regulation theory, individuals who lack a sense of security are more likely to have negative expectations about future events and heightened emotional vigilance, manifesting as elevated levels of generalized anxiety and emotional dysregulation (Fonagy and Luyten, 2009). In confined and high-pressure prison environments, those with a lower sense of security tend to exhibit anxiety-related symptoms such as emotional outbursts, hypervigilance, and impulsivity (Liebling and Arnold, 2004; McTernan et al., 2023).

Third, emotional problems are widely recognized as factors significantly associated with NSSI of prisoners (Favril et al., 2020; McTernan et al., 2023). Researchers have found that when individuals experience negative emotion overload, particularly intense anxiety and depression, they may engage in self-harm as a short-term emotion regulation strategy to escape, alleviate, or “numb” negative emotional experiences (Klonsky, 2007). Since incarcerated populations have limited access to adaptive coping resources, NSSIs may be an accessible yet inappropriate way for individuals to experience a sense of “control” or “release,” while serving as a means of protesting prison management and seeking relief or assistance (Dear et al., 2000; Perry, 2020).

**Figure 1:**
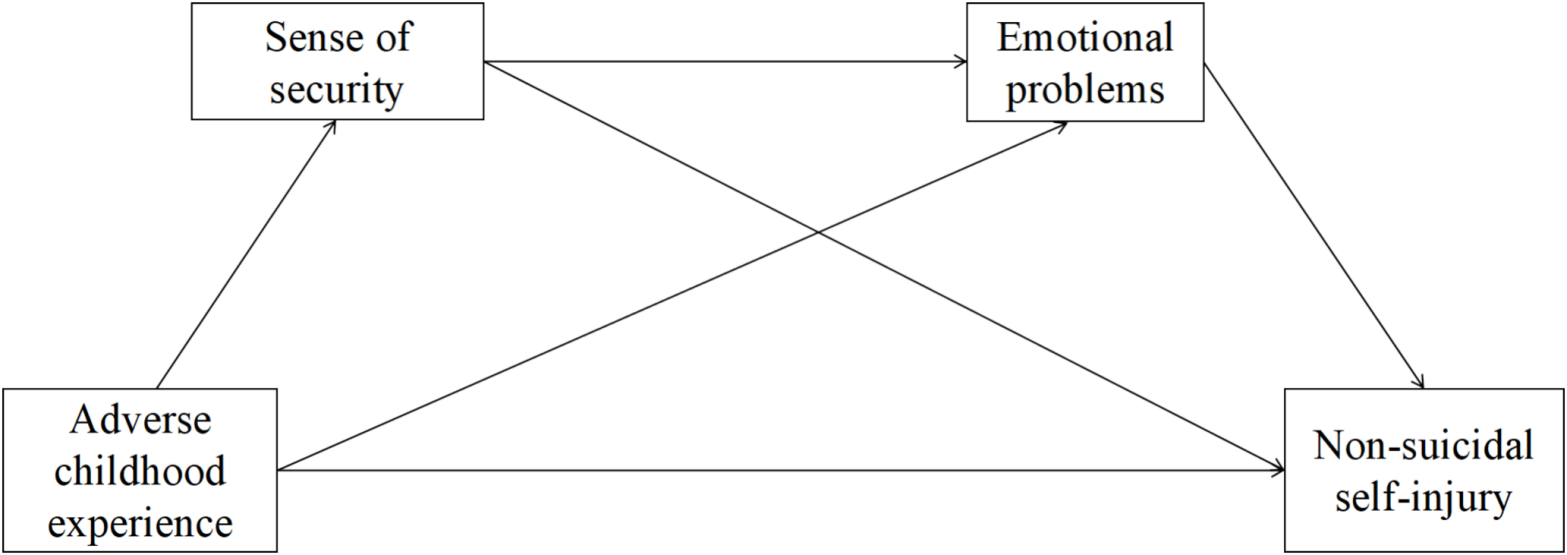
The hypothesis model for this study.

To sum up, we aimed to examine the relationships among ACE, sense of security, emotional problems, and NSSI in male prisoners. A chain mediation model was developed to investigate both direct and indirect pathways between these variables. The primary objective was to explore potential antecedents and underlying mechanisms of NSSI in this population, with the goal of providing empirical support to prevent NSSI in correctional settings. The hypothesized relationships between the variables are illustrated in Figure 1. Four hypotheses were formulated: (1) ACE, sense of security, emotional problems, and NSSI are significantly interrelated; (2) Sense of security mediates the relationship between ACE and NSSI for male prisoners; (3) Emotional problems mediate the relationship between ACE and NSSI for male prisoners; (4) There is a sequential mediating effect of sense of security and emotional problems in the association between ACE and NSSI for male prisoners.

## 2 Materials and methods

### 2.1 Participants

Data were collected from male prisoners incarcerated in two prisons in a southeastern province of China. Prison officers from these two male-only prisons were asked to relay information about the study and invite volunteers to participate. Before data collection, participants were informed that their decision to participate would not affect their regular management and routine care, and that they could withdraw from the study at any time without consequence. Prisoners who agreed to participate signed an informed consent form and completed the relevant questionnaires. Additionally, participants were informed that they could file complaints regarding their participation if they wished. Prison staff had no access to individual scores, and the data used for the study consisted solely of self-reported information.

Our study was approved by the Ethics Committee of Fujian Police College for research involving human subjects and was conducted in full compliance with the 1964 Declaration of Helsinki and its later addenda. The approval number is FPCER-202404-003. The participants were informed of the study’s purposes and procedures. All gave their written informed consent before the study commenced.

A total of 1,205 incarcerated individuals were invited to complete a demographic survey, along with questionnaires assessing NSSI, ACEs, sense of security, and emotional problems. Of these, 472 male prisoners reported a history of NSSI behavior, yielding an initial detection rate of 39.17%. After data review, 74 questionnaires were excluded due to dishonest, evasive or invalid responses, resulting in 398 valid questionnaires, and an actual NSSI detection rate of 33.03%. The demographic variables of the 398 male prisoners who reported NSSI, who had a mean age of 35.90 years (SD = 8.70), are presented in Table 1, below. The mean sentence length, as determined by Chinese courts, was 7.96 years (SD = 5.12), while the mean time already served in prison at the time of the study was 4.70 years (SD = 3.61).

**Table 1:** Demographics of participants (*N* = 398).

Characteristics	Frequency
Age	
18–30	113 (28.4%)
31–40	185 (46.5%)
41–50	68 (17.1%)
≥51	32 (8.0%)
Educational level	
≤Junior high	49 (12.3%)
Senior high	73 (18.3%)
Undergraduate	265 (66.6%)
≥Postgraduate	11 (2.8%)
First-time offender	
Yes	229 (57.5%)
No	169 (42.5%)
Drug use history	
Yes	85 (21.4%)
No	313 (78.6%)

The inclusion criteria were as follows: (1) participants had to take part voluntarily and give their written informed consent; (2) they needed sufficient cognitive ability to complete the questionnaires; (3) they had to be aged between 18 and 75. Prisoners deemed ineligible by prison staff were not invited to participate, either due to failure to meet the inclusion criteria or because their mental or physical state was deemed unsuitable for participation during the completion of the questionnaires.

### 2.2 Measures

#### 2.2.1 Adverse childhood experiences (ACEs)

We used the Chinese version of the Adverse Childhood Experiences International Questionnaire (ACE-IQ) to assess ACEs (World Health Organization, 2018), which has been confirmed to have good reliability and validity (Ho et al., 2019; Mi et al., 2023). The ACE-IQ is a self-report scale comprising 29 items and divided into three main dimensions: (1) Childhood maltreatment, which includes items such as “Did a parent, guardian or other household member yell, scream or swear at you, insult or humiliate you?”; (2) Family/ Household dysfunction, such as “Did you live with a household member who was depressed, mentally ill or suicidal?”; (3) Violence outside the home (e.g., at school, in the neighborhood, or society), such as “Was a family member or friend killed or beaten up by soldiers, police, militia or gangs?.” Response options for each question include dichotomous (i.e., Yes/ No), 5-point Likert scales ranging from “Never” to “Always,” or a 4-point Likert scale ranging from “Never” to “Many times.” Higher ACE-IQ scores indicate more severe ACEs. In this study, the Cronbach’s α coefficient for the ACE-IQ was 0.882, and the McDonald’s *ω* was 0.886.

#### 2.2.2 Sense of security

We used the Security Questionnaire (SQ) to assess sense of security, which has good reliability and validity (Cong and An, 2004; Liao et al., 2014). The SQ is a self-report scale comprising 16 items that measure two factors: interpersonal security (e.g., “I always worry that close friendships will deteriorate in the future”) and certainty of control (e.g., “I feel that life is always filled with uncertainty and unpredictability”). To ensure consistency in the direction of variable changes, the SQ scores were reversed in our study. Thus, higher scores indicate a lower sense of security. Responses are rated on a 5-point Likert scale, ranging from 1 to 5. In this study, the Cronbach α for the SQ was 0.913 and the McDonald’s ω was 0.914.

#### 2.2.3 Emotional problems

We used the Chinese version of the Irritability, Depression, and Anxiety Scale (IDA) to assess emotional problems in male prisoners (Snaith and Taylor, 1985; Yuan et al., 2002). This version includes 18 items that assess four dimensions: (1) Depression, with 5 items, such as “I can laugh and feel happy”; (2) Anxiety, with 5 items, such as “I feel panic or worry for no reason”; (3) Outward irritability, with 4 items, such as “People annoy me so much that I want to break doors or throw things”; (4) Inward irritability, with 4 items, such as “I get upset with myself or feel irritated when I hear my name.” Responses are rated based on the actual condition, ranging from no symptoms to severe symptoms (with some items reverse-scored), on a scale of 0 to 3. Higher scores on the IDA indicate more severe corresponding symptoms. In this study, the Cronbach *α* for the IDA was 0.833, and the McDonald’s *ω* was 0.838.

#### 2.2.4 Non-suicidal self-injury (NSSI)

We assessed NSSI using the Adolescent Non-suicidal Self-injury Assessment Questionnaire (ANSAQ), a tool developed by Wan et al. (2018). Although originally designed for adolescents, the ANSAQ has been validated in adult populations, with authors of existing studies confirming its reliability and psychometric robustness for adults (Lin and Jiang, 2025; Lu et al., 2024).

The ANSAQ has two subscales: Behavioral and Functional Questionnaires. The Behavioral Questionnaire includes 12 items (e.g., “I deliberately cut myself using objects like a knife blade or glass”) using a 5-point Likert scale. Each item is rated from 0 to 4, corresponding to the following options: “Never” (0), “Rarely” (1), “Sometimes” (2), “Most of the times” (3), and “Always” (4). The Functional Questionnaire consists of 19 items (e.g., “Cope with feelings of sadness or disappointment”), using a 5-point Likert scale. Each item is rated from 0 to 4, corresponding to the following options: “Greatly disagree” (0), “Disagree” (1), “Uncertain” (2), “Agree” (3), and “Greatly agree” (4).

Participants who did not exhibit any NSSI behavior (as indicated by a score of zero on the Behavioral Questionnaire) were not required to complete the Functional Questionnaire. In this study, the Cronbach’s α coefficient was 0.912 for the Behavioral Questionnaire, 0.946 for the Functional Questionnaire, and 0.941 for the entire scale. The McDonald’s ω was 0.913 for the Behavioral Questionnaire, 0.946 for the Functional Questionnaire, and 0.946 for the entire scale.

### 2.3 Statistical analysis

We conducted data analysis using SPSS 24.0 and Amos 26.0. First, descriptive statistics were calculated in SPSS 24.0, and the correlations among variables were examined. Subsequently, a structural equation model was constructed using Amos 26.0 to test the serial mediation effect of SQ and IDA scores on the relationship between ACEs and NSSI for male prisoners.

### 2.4 Common method biases

In order to eliminate the common method deviation caused by the questionnaire survey, we used the Harman single-factor test. After performing the principal component analysis (PCA), 21 eigenvalues greater than 1 were extracted. The explained variance percentage of the first common factor was 19.96% (less than 40%), which indicates that the current study did not have any problems with common method biases.

## 3 Results

### 3.1 Descriptive statistics and correlation

The descriptive statistics for the dimensions assessed in this study can be seen in Table 2. Since the distribution of total NSSI scores failed the examination of normality assumption (K-S statistic = 0.057, *p* = 0.004), a normalizing transformation (case ranking method) was applied to the total score and two factor scores of NSSI to meet the requirements for parametric analyses.

**Table 2:** Descriptive statistics (*N* = 398).

Variable	Mean	Std. deviation
ACE1 (childhood maltreatment)	0.599	0.347
ACE2 (family/household dysfunction)	0.272	0.306
ACE3 (violence outside the home)	0.622	0.518
ACE (total score)	0.513	0.320
SQ1 (interpersonal security)	2.633	0.790
SQ2 (certainty in control)	2.967	0.828
SQ (total score)	2.799	0.765
IDA1 (depression)	1.255	0.524
IDA2 (anxiety)	1.342	0.486
IDA3 (outward irritability)	1.010	0.674
IDA4 (inward irritability)	1.186	0.546
IDA (total score)	1.209	0.427
NSSI1 (self-injurious behavior)	0.342	0.490
NSSI2 (self-injurious function)	1.349	0.819
NSSI (total score)	0.954	0.582

ACE, adverse childhood experience; SQ, security questionnaire; IDA, Irritability, Depression, and Anxiety Scale; NSSI, non-suicidal self-injury.

The correlation matrix results in Table 3 demonstrate that the total score and three factors of ACE were significantly correlated with the self-injurious behavior factor of NSSI (*p* < 0.01), as well as the self-injurious function factor (*p* < 0.01). The total score and two SQ factors were significantly associated with ACE and NSSI. The four IDA factors were correlated with ACE, SQ and NSSI total and dimensional scores (*p* < 0.01). These correlation results provide preliminary support for the associations between the variables proposed in the study hypotheses.

**Table 3:** Correlation matrix between factors (*N* = 398).

Variable	ACE1	ACE2	ACE3	ACE	SQ1	SQ2	SQ	IDA1	IDA2	IDA3	IDA4	IDA	NSSI1	NSSI2
ACE2	0.573^**^													
ACE3	0.513^**^	0.540^**^												
ACE	0.865^**^	0.782^**^	0.838^**^											
SQ1	0.361^**^	0.259^**^	0.213^**^	0.338^**^										
SQ2	0.361^**^	0.279^**^	0.336^**^	0.399^**^	0.789^**^									
SQ	0.382^**^	0.284^**^	0.292^**^	0.390^**^	0.943^**^	0.948^**^								
IDA1	0.186^**^	0.163^**^	0.275^**^	0.256^**^	0.301^**^	0.296^**^	0.316^**^							
IDA2	0.143^**^	0.191^**^	0.306^**^	0.256^**^	0.388^**^	0.459^**^	0.448^**^	0.468^**^						
IDA3	0.332^**^	0.327^**^	0.362^**^	0.408^**^	0.469^**^	0.548^**^	0.539^**^	0.479^**^	0.545^**^					
IDA4	0.225^**^	0.174^**^	0.352^**^	0.312^**^	0.314^**^	0.377^**^	0.366^**^	0.350^**^	0.363^**^	0.575^**^				
IDA	0.288^**^	0.279^**^	0.416^**^	0.399^**^	0.478^**^	0.545^**^	0.541^**^	0.755^**^	0.769^**^	0.848^**^	0.719^**^			
NSSI1	0.415^**^	0.364^**^	0.398^**^	0.475^**^	0.263^**^	0.328^**^	0.313^**^	0.301^**^	0.261^**^	0.440^**^	0.338^**^	0.434^**^		
NSSI2	0.255^**^	0.175^**^	0.238^**^	0.276^**^	0.271^**^	0.331^**^	0.319^**^	0.203^**^	0.258^**^	0.413^**^	0.347^**^	0.393^**^	0.365^**^	
NSSI	0.283^**^	0.198^**^	0.272^**^	0.311^**^	0.172^**^	0.194^**^	0.194^**^	0.156^**^	0.119^*^	0.237^**^	0.210^**^	0.233^**^	0.533^**^	0.268^**^

ACE: adverse childhood experience; SQ: security questionnaire; IDA: Irritability, Depression, and Anxiety Scale; NSSI: non-suicidal self-injury. ^**^Correlation is significant at the 0.01 level. ^*^Correlation is significant at the 0.05 level.

### 3.2 Model fit for structural equation modeling

Two serial mediation models were constructed in this study to examine the mediating roles of security sense and emotional problems in the relationship between ACE and NSSI among male prisoners, using the NSSI Behavior or Function Questionnaire scores as outcome variables in each model, respectively.

**Figure 2:**
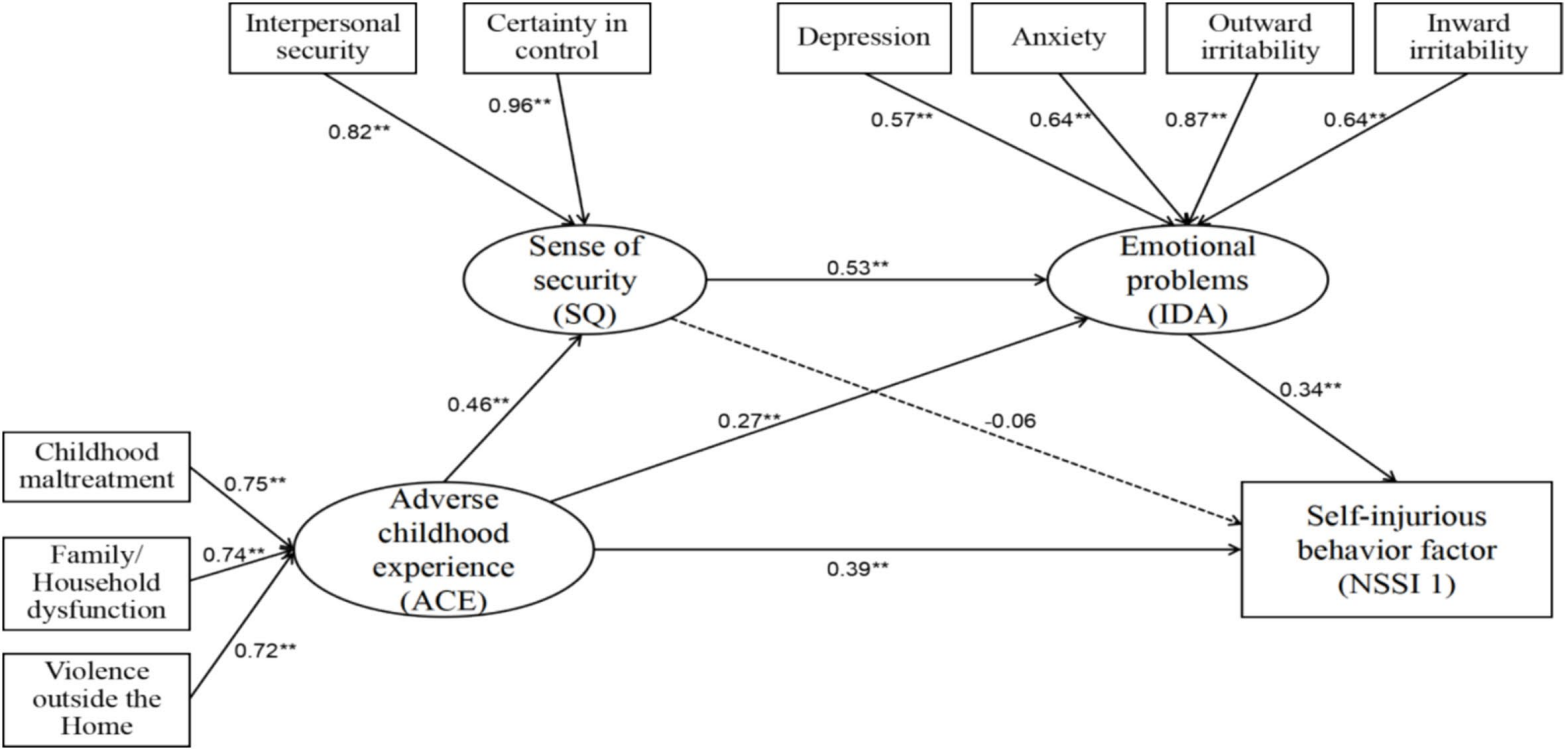
Standardized path coefficients of Model A. The numbers attached to single-headed arrows reflect the standardized path efficiency. *p* < 0.01.

The model fit indices for Model A, with the NSSI Behavior Questionnaire score as the outcome variable, can be seen in Table 4 and Figure 2. The chi-square value was 98.586 (df = 30, *p* < 0.001), and the *χ*^2^/df was 3.286. The ratio of chi-square to degrees of freedom values was <5, indicating that the model is acceptable (Schumacker and Lomax, 2004; Tong and Bentler, 2013). The Comparative Fit Index (CFI) was 0.957, surpassing the good fit criterion (≥0.90). The Tucker-Lewis Index (TLI) was 0.935 and the Normed Fit Index (NFI) was 0.940, both exceeding 0.90, indicating good model fit. The Root Mean Square Error of Approximation (RMSEA) was 0.076, indicating acceptable fit (≤0.08) (Hu and Bentler, 1999; McDonald and Ho, 2002). The Standardized Root Mean Square Residual (SRMR) was 0.040, which was below the 0.08 threshold and so indicated small residuals. The Goodness of Fit Index (GFI) was 0.955 and the Adjusted Goodness of Fit Index (AGFI) was 0.917, both of which exceeded 0.90, further supporting good model fit. Taken together, these indices suggest that Model A had an acceptable fit and was suitable for subsequent parameter estimation and mediation effect analysis.

**Table 4:** Goodness-of-fit index of the structural Model A.

Fit indices	Criteria	Model fit of the research model
χ^2^	The smaller, the better	98.586
df	The larger, the better	30
Normed chi-square (*χ*^2^/df)	1 < *χ*^2^/df < 5	3.286
RMSEA	<0.08	0.076
SRMR	<0.08	0.040
TLI	>0.9	0.935
NFI	>0.9	0.940
CFI	>0.9	0.957
GFI	>0.9	0.955
AGFI	>0.9	0.917
IFI	>0.9	0.957

*χ*^2^, Chi-square; df, degrees of freedom; RMSEA, root mean square error of approximation; SRMR: standardized root mean square residual; TLI: Tucker-Lewis Index; NFI: Normed Fit Index; CFI: Comparative Fit Index; GFI: Goodness-of-Fit Index; AGFI: Adjusted Goodness-of-Fit Index; IFI: Incremental Fit Index.

**Figure 3:**
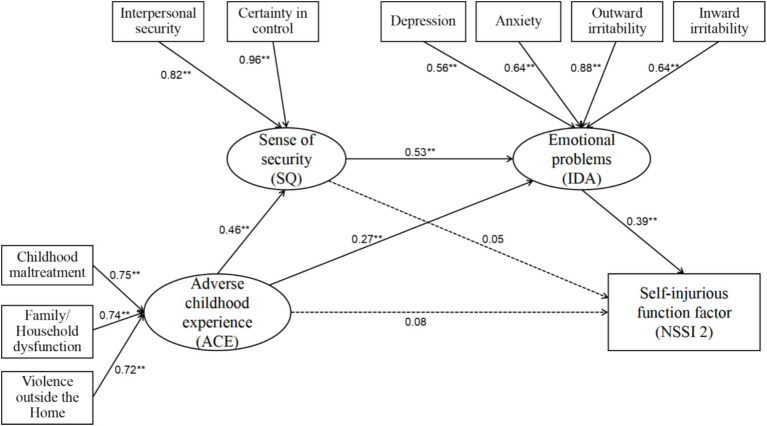
Standardized path coefficients of Model A. The numbers attached to the single-headed arrows reflect the standardized path efficiency. *p* < 0.01.

The model fit indices for Model B, with the NSSI function questionnaire score as the outcome variable, can be seen in Table 5 and Figure 3. The chi-square value was 101.237 (df = 30, *p* < 0.001), and the *χ*^2^/df was 3.375. The CFI was 0.954, surpassing the good fit criterion (≥0.90). The TLI was 0.930 and the NFI was 0.936, indicating good model fit. The RMSEA was 0.077 and the SRMR was 0.042, below the 0.08 threshold. The GFI was 0.953 and the AGFI was 0.914, both indicating good model fit. Therefore, these indices suggest that Model B had an acceptable fit and was suitable for further parameter estimation and mediation effect analysis.

**Table 5:** Goodness-of-fit index of the structural Model B.

Fit indices	Criteria	Model fit of the research model
*χ* ^2^	The smaller, the better	101.237
df	The larger, the better	30
Normed Chi-square (*χ*^2^/df)	1 < *χ*^2^/df < 5	3.375
RMSEA	<0.08	0.077
SRMR	<0.08	0.042
TLI	>0.9	0.930
NFI	>0.9	0.936
CFI	>0.9	0.954
GFI	>0.9	0.953
AGFI	>0.9	0.914
IFI	>0.9	0.954

*χ*^2^, Chi-square; df, degree of freedom; RMSEA, root mean square error of approximation; SRMR, standardized root mean square residual; TLI, Tucker-Lewis Index; NFI, Normed Fit Index; CFI, Comparative Fit Index; GFI, Goodness-of-Fit Index; AGFI, Adjusted Goodness-of-Fit Index; IFI, Incremental Fit Index.

### 3.3 Mediating effects analysis

#### 3.3.1 Direct and indirect effect of Model A

The effect estimates for each direct and indirect pathway in Model A can be seen in Tables 6, 7. The direct effect of SQ on self-injurious behavior was not significant (*p* = 0.346), and neither was the simple mediation effect of ACE on self-injurious behavior through SQ (*p* = 0.353). The serial mediation effect of ACE on self-injurious behavior through SQ and IDA was significant (*p* = 0.002), suggesting that ACE is associated with a diminished sense of security, which is in turn associated with more emotional problems, and these factors together are linked to more frequent self-injurious behaviors. The simple mediation effect of ACE on self-injurious behavior through IDA was also significant (*p* = 0.005), indicating that IDA plays a significant mediating role in the relationship between ACE and self-injurious behaviors. The total indirect effect was significant (*p* = 0.001). Together with the significant direct effect (*p* = 0.002), these constitute the total effect (*p* = 0.002), demonstrating that ACE influences NSSI behaviors through multiple pathways.

**Table 6:** Estimate of direct pathways of Model A.

Path	Estimate	SE	C.R.	*p*
ACE→SQ	0.811	0.114	7.114	<0.001
SQ → IDA	0.242	0.032	7.592	<0.001
ACE→IDA	0.213	0.051	4.214	<0.001
IDA → NSSI 1 (behavior)	1.016	0.229	4.438	<0.001
SQ → NSSI 1 (behavior)	−0.084	0.089	−0.943	0.346
ACE→NSSI 1 (behavior)	0.931	0.154	6.041	<0.001

ACE, adverse childhood experience; SQ, security questionnaire; IDA, irritability, depression, and anxiety; NSSI, non-suicidal self-injury.

**Table 7:** Estimate of indirect pathways of Model A.

Path	Estimate	SE	Z	Bias-corrected percentile		
				Lower	Upper	*p*
ACE → SQ → NSSI 1 (behavior)	−0.068	0.090	−0.756	−0.279	0.072	0.353
ACE → IDA → NSSI 1 (behavior)	0.216	0.088	2.455	0.071	0.431	0.005
ACE → SQ → IDA → NSSI 1 (behavior)	0.200	0.086	2.326	0.078	0.42	0.002
Total indirect effect	0.348	0.098	3.551	0.181	0.577	0.001
Direct effect	0.931	0.180	5.172	0.61	1.328	0.002
Total effect	1.279	0.136	9.404	1.035	1.578	0.002

ACE, adverse childhood experience; SQ, security questionnaire; IDA, irritability, depression, and anxiety; NSSI, non-suicidal self-injury. Bias-corrected percentile: confidence interval 95%; bootstrap 1,000 times.

#### 3.3.2 Direct and indirect effect of Model B

The effect estimates for each direct and indirect pathway in Model B can be seen in Tables 8, 9. The direct effect of SQ on self-injurious function was not significant (*p* = 0.434), and neither was the simple mediation effect of ACE on self-injurious function through SQ (*p* = 0.470). The direct effect of ACE on self-injurious function was not significant (*p* = 0.225). The serial mediation effect of ACE on self-injurious function through SQ and IDA was significant (*p* = 0.001), suggesting that ACE is associated with the increased maladaptive function of NSSI through sense of security impairment and emotional problems. The simple mediation effect of ACE on self-injurious function through IDA was also significant (*p* = 0.006), indicating that IDA plays a significant mediating role in the relationship between ACE and self-injurious function. The total indirect effect is statistically significant (*p* = 0.003), as is the total effect (*p* = 0.002).

**Table 8:** Estimate of direct pathways of Model B.

Path	Estimate	SE	C.R.	*p*
ACE→SQ	0.804	0.114	7.078	<0.001
SQ → IDA	0.238	0.032	7.537	<0.001
ACE→IDA	0.211	0.050	4.241	<0.001
IDA → NSSI 2 (function)	1.295	0.277	4.670	<0.001
SQ → NSSI 2 (function)	0.081	0.104	0.782	0.434
ACE→NSSI 2 (function)	0.207	0.170	1.214	0.225

ACE, adverse childhood experience; SQ, security questionnaire; IDA, irritability, depression, and anxiety; NSSI, non-suicidal self-injury.

**Table 9:** Estimate of indirect pathways of Model B.

Path	Estimate	SE	Z	Bias-corrected percentile		
				Lower	Upper	*p*
ACE → SQ → NSSI 2 (function)	0.065	0.101	0.644	−0.117	0.290	0.470
ACE → IDA → NSSI 2 (function)	0.273	0.128	2.133	0.065	0.563	0.006
ACE → SQ → IDA → NSSI 2 (function)	0.248	0.091	2.725	0.122	0.496	0.001
Total indirect effect	0.586	0.130	4.508	0.349	0.850	0.003
Direct effect	0.207	0.188	1.101	−0.136	0.562	0.283
Total effect	0.792	0.158	5.013	0.487	1.089	0.002

ACE, adverse childhood experience; SQ, security questionnaire; IDA, irritability, depression, and anxiety; NSSI, non-suicidal self-injury. Bias-corrected percentile: confidence interval 95%; bootstrap 1,000 times.

## 4 Discussion

Although previous studies have extensively discussed the association between ACEs and NSSI (Gu et al., 2021; Hariz et al., 2025; Laporte et al., 2023; Li et al., 2024; Seibert, 2012; Takahashi et al., 2022; Van Heeringen, 2018), this study is the first, to the best of our knowledge, to investigate the serially mediating effect of sense of security and emotional problems between ACEs and NSSI among prisoners. Additionally, this study is the first to examine the indirect pathways from ACEs to two dimensions of NSSI (behaviors and functions), among male prisoners. We found that ACEs are both directly and indirectly associated with NSSI behavioral factor among male prisoners, while their association with NSSI functional factor is solely indirect.

These results are significant for early psychological intervention in prisoners and for the identification of NSSI risks, thereby contributing to improved prison management. They also provide insights into the different mechanisms by which ACEs are linked to NSSI behaviors and functions. The results will be discussed in the following three sections.

### 4.1 Correlation between variables

As expected, significant correlations were found between ACEs, sense of security, IDA, and NSSI among male prisoners. Specifically, ACEs were significantly positively correlated with NSSI in incarcerated male offenders, which is consistent with previous studies on NSSI behaviors in prisoners (Gu et al., 2021; Hariz et al., 2025; Li et al., 2024; Takahashi et al., 2022). Stinson et al. (2021) noted that ACEs are prevalent among male prisoners and are significantly associated with NSSI behaviors. In their review, Favril et al. (2020) confirmed that male prisoners with more ACEs tend to experience more thoughts related to NSSI, which is an important indicator of potential suicide attempts within prisons. These findings, in conjunction with those from this study, suggest that prisoners who have had more ACEs should be given more attention and access to resources for preventing self-harm and suicide risk, especially given the current situation, where mental health resources within prisons are minimal (Fazel et al., 2016; Hill et al., 2022).

Based on our results, sense of security is significantly correlated with NSSI in male prisoners. Deng et al. (2024) found that sense of security is strongly associated with suicidal ideation in a prison environment, aligning with research on sense of security and suicide risk in male prisoners (Wang and Sun, 2024). This suggests that sense of security is uniquely associated with NSSI. Finally, a significant correlation was found between emotional problems and NSSI in male prisoners, consistent with previous research (Gu et al., 2021; Taja et al., 2025). This highlights that anxiety, depression, and irritability remain significant factors related to NSSI in incarcerated individuals.

### 4.2 Direct and indirect effects of ACEs on NSSI

For the participants in this study, childhood maltreatment (ACE 1), family/household dysfunction (ACE 2), and violence outside the home (ACE 3) were all significantly correlated with NSSI. This underscores the long-term association between ACEs and the psychological health and behavioral risks of male prisoners. Our findings suggest that these early adverse experiences may lay a psychological foundation for the subsequent development of self-injurious behaviors. This is consistent with previous research on offender populations, where it has been shown that ACEs are not only associated with an increased risk of emotional disorders in prisoners but also significantly linked to higher incidences of NSSI (Gu et al., 2023; Seibert, 2012).

Notably, the results of our model examination indicate that ACEs have both direct and indirect effects on NSSI behavior, while having only an indirect effect on the functional aspect of NSSI. These results can be explained in two ways.

On the one hand, according to proponents of social learning theory (Bandura and Walters, 1977), individuals may acquire self-harming behaviors from ACEs (whether they were abused by others or witnessed others engaging in violent behaviors). This effect has been documented in studies on the relationship between ACEs and suicide behavior (Van Heeringen, 2018). Specifically, exposure to abuse is associated with an increased likelihood of individuals learning and imitating such behaviors, making them more feasible. Therefore, ACEs exhibit both a direct association with NSSI behavior and an indirect relationship mediated by factors such as sense of security and emotional difficulties.

On the other hand, experiences of childhood abuse and neglect are often inadequately addressed, with unresolved trauma potentially being associated with the use of self-injury as a means of emotional regulation (Knight et al., 2017). Childhood maltreatment is associated with alterations in an individual’s interpersonal information processing, which in turn is linked to a heightened propensity for hostile attribution bias—that is, the tendency to perceive others’ behaviors as hostile or defensive—and this bias is further associated with difficulties in interpersonal adjustment and mental health problems (Dodge et al., 1995). In the high-pressure environment of prisons, such cognitive biases may be more pronounced, and individuals with these biases may be more likely to choose self-injury as a non-verbal and maladaptive outlet for emotional release when confronted with internal distress and external pressures. Therefore, ACEs are more likely to show an indirect effect than a direct effect on the NSSI function on prisoners.

### 4.3 The serial mediation role of sense of security and emotional problems

A significant serial mediation pathway was found in this study, through which the sense of security and IDA mediate the relationship between ACEs and NSSI (both behavior and function) in male prisoners. These pathways uncover the psychological mechanism linking early trauma to NSSI in adults, suggesting that ACEs are associated with a diminished sense of security in individuals, which is in turn associated with more severe emotional problems and indirectly linked to an elevated risk of self-injury.

According to proponents of life history theory, couched within an evolutionary framework (Liu, 2019; Belsky et al., 2012; Del Giudice et al., 2011; Ellis et al., 2009), if individuals do not secure basic environmental safety and predictability during their development, they are more likely to perceive the external world as threatening and uncertain. Psychologists with a trauma-healing approach believe that excessive vigilance and sensitivity to negative information, as well as overreactions, are survival strategies that stem from ACEs (Herman, 1997; Walker, 2013). This persistent sense of insecurity is associated with the development of hostility and distrust in individuals, which is linked to reduced internal emotional stability resources. Neuroscientists have pointed out that the significant mediating effect of emotional problems between ACEs and NSSI can, in part, be linked to the lasting effects of ACEs, such as the over-activation of the HPA axis and functional imbalances in brain areas related to emotional regulation, such as the amygdala and prefrontal cortex (Hughes et al., 2017; Teicher and Samson, 2016).

Additionally, the unattainability of a sense of security is an inevitable condition and prevailing atmosphere within prison settings, which is associated with heightened sensitivity to external threats. Therefore, prisoners’ already fragile emotional regulation processes, such as cognitive reappraisal and response modulation (Gross, 2001, 2015), may be more vulnerable to failure. In this context, incarcerated individuals become more prone to emotional problems such as irritability, depression, and anxiety, which are associated with an increased likelihood of NSSI behavior frequency.

## 5 Implications and limitations

Although researchers have extensively explored the relationship between ACEs and NSSI in offender populations (Hariz et al., 2025; Li et al., 2024; Takahashi et al., 2022), we examined the serial mediating roles of sense of security and emotional problems in the relationship between ACEs and NSSI in prisoners. Our findings enhance understanding of the relationship between ACEs and NSSI in incarcerated individuals. Furthermore, this serial mediation model is comprehensive, empirical support for the need to develop interventions aimed at reducing NSSI among prison inmates, including those that address ACEs, enhance prisoners’ sense of security, and improve emotional regulation.

This study has certain limitations that must be acknowledged. First, the analytical sample was limited to prisoners who reported engaging in NSSI behaviors, which limits the generalizability and scope of the findings. These results cannot be interpreted as direct evidence of factors predicting the risk of initiating NSSI behavior among incarcerated individuals without a prior history of self-injury. Although we found that ACEs, reduced sense of security, and emotional problems are associated with more severe NSSI, future research should employ a case–control design—comparing prisoners with and without NSSI—or prospective cohort studies to examine factors related to the onset of NSSI. Such designs would yield more direct evidence for risk prediction and primary prevention. Second, we relied on self-report questionnaires to collect quantitative data, which may introduce social desirability bias. For example, some prisoners may have avoided reporting NSSI behaviors, ACEs, or their emotional distress, potentially leading to distorted data. To mitigate this issue, future researchers could adopt a mixed-methods approach, triangulating data from multiple sources to provide a richer, more detailed understanding of NSSI behaviors in prisoners. Third, the cross-sectional design of this study limits the ability to make causal inferences. To explore temporal dynamics and establish causal relationships, future researchers exploring NSSI in prisoners could use a longitudinal design, such as a cross-lagged panel model, to capture how variables interact over time. This could lead to a more comprehensive understanding of the mechanisms underlying the effects of various variables at different time points. Finally, as for many studies in criminology (Gu et al., 2023; Morales and Guarnero, 2014), we did not include a sample of female prisoners, which may limit the generalizability of our findings. Therefore, future research should include samples of female prisoners.

## 6 Conclusion

This study has verified the correlations among ACEs, sense of security, emotional problems, and NSSI in prisoners, and further demonstrated how ACEs, sense of security, and emotional problems interact in a pathway linked to NSSI behaviors and functions. ACEs are associated with NSSI behaviors through both direct pathways and via a chain-mediated pathway through a sense of security and emotional problems. However, ACEs are only indirectly related to NSSI functions. Interventions aimed at improving the handling of childhood adversities and trauma recovery, enhancing prison management to increase inmates’ sense of security, and introducing emotional regulation training programs could help mitigate the effect of ACEs on NSSI in male prisoners, thereby potentially reducing the incidence of NSSI in correctional settings.

## Data Availability

The raw data supporting the conclusions of this article will be made available by the authors, without undue reservation.
